# Supplementary material for: Virtual reality for stress management and burnout reduction in nursing: A systematic review protocol
Source: PLoS One. 2025 Apr 7;20(4):e0319247. doi: 10.1371/journal.pone.0319247 (PMC11975074; doi:10.1371/journal.pone.0319247)
Supplement: S1 File — (DOC) [file pone.0319247.s002.docx]

**Appendix 1. Data extraction form**

| **Categories** | **Variables** |
| --- | --- |
| **Study ID** | - First author  - Publication year  - Country  - Funding source  - Conflict of interest |
| **Methods** | - Study design (e.g., RCT, quasi-experimental, pre-post)  - Study setting (e.g., hospital, long-term care)  - Participant recruitment and sampling  - Inclusion and exclusion criteria  - Sample size and power analysis  - Randomization and allocation concealment (if applicable)  - Blinding of participants and personnel (if applicable)  - Comparator group (if applicable)  - Length of follow-up |
| **Participants** | - Sample size  - Age (mean, SD, range)  - Gender (n, %)  - Race/ethnicity (n, %)  - Nursing specialty (n, %)  - Years of nursing experience (mean, SD, range)  - Baseline stress and burnout scores (mean, SD) |
| **Intervention** | - VR equipment and platform  - VR content and environment  - Therapeutic approach and theoretical basis  - Dose, frequency, and duration of VR sessions  - Setting and context of delivery  - Facilitators and barriers to delivery  - Fidelity and adherence measures  - Adverse events or side effects |
| **Outcomes** | - Measures of stress (self-report, physiological, behavioral)  - Measures of burnout (self-report)  - Secondary and exploratory outcomes  - Timing of assessments  - Effect sizes and 95% confidence intervals  - Within-group and between-group comparisons  - Subgroup and sensitivity analyses  - Handling of missing data and attrition |
| **Conclusions** | - Key findings and interpretations  - Strengths and limitations  - Implications for practice and research  - Suggested future directions |

**Appendix 2. Risk of bias assessment tools**

**Cochrane Risk of Bias 2 (RoB 2) tool for randomized trials**

| **Domain** | **Questions** |
| --- | --- |
| Randomization process | Was the allocation sequence random? Was the allocation sequence concealed until participants were enrolled and assigned to interventions? Did baseline differences between intervention groups suggest a problem with the randomization process? |
| Deviations from intended interventions | Were participants aware of their assigned intervention during the trial? Were carers and people delivering the interventions aware of participants' assigned intervention during the trial? Were there deviations from the intended intervention that arose because of the trial context? Were these deviations likely to have affected the outcome? Was an appropriate analysis used to estimate the effect of assignment to intervention? Was there potential for a substantial impact of the failure to analyse participants in the group to which they were randomized? |
| Missing outcome data | Were outcome data available for all, or nearly all, participants randomized? Is there evidence that result was not biased by missing outcome data? Could missingness in the outcome depend on its true value? Is it likely that missingness in the outcome depended on its true value? |
| Measurement of the outcome | Was the method of measuring the outcome inappropriate? Could measurement or ascertainment of the outcome have differed between intervention groups? Were outcome assessors aware of the intervention received by study participants? Could assessment of the outcome have been influenced by knowledge of intervention received? |
| Selection of the reported result | Was the trial analysed in accordance with a pre-specified plan that was finalized before unblinded outcome data were available for analysis? Is the numerical result being assessed likely to have been selected, on the basis of the results, from multiple outcome measurements within the outcome domain? Is the numerical result being assessed likely to have been selected, on the basis of the results, from multiple analyses of the data? |

Responses: Yes, Probably yes, Probably no, No, No information

**Risk of Bias in Non-randomized Studies of Interventions (ROBINS-I) tool**

| **Domain** | **Questions** |
| --- | --- |
| Bias due to confounding | Is there potential for confounding of the effect of intervention in this study? Were confounding domains that were controlled for measured validly and reliably by the variables available in this study? Were confounding domains that were controlled for implemented effectively? Was confounding potentially a problem? |
| Bias due to selection of participants | Was selection of participants into the study (or into the analysis) based on participant characteristics observed after the start of intervention? Do start of follow-up and start of intervention coincide for most participants? Were adjustment techniques used that are likely to correct for the presence of selection biases? Is the selection into the study likely unrelated to intervention or unrelated to outcome? Do start of follow-up and start of intervention coincide? Were the post-intervention variables that influenced selection likely to be associated with intervention? Was the selection into the study likely related to intervention or outcome? |
| Bias in classification of interventions | Is intervention status well defined? Was information on intervention status recorded at the time of intervention? Was information on intervention status unaffected by knowledge of the outcome or risk of the outcome? Is classification of intervention status likely to be affected by knowledge of the outcome or risk of the outcome? |
| Bias due to deviations from intended interventions | Were there deviations from the intended intervention beyond what would be expected in usual practice? Were these deviations from intended intervention unbalanced between groups and likely to have affected the outcome? Were important co-interventions balanced across intervention groups? |
| Bias due to missing data | Were outcome data available for all, or nearly all, participants? Were participants excluded due to missing data on intervention status? Were participants excluded due to missing data on other variables needed for the analysis? Are the proportion of participants and reasons for missing data similar across interventions? Is there evidence that results were robust to the presence of missing data? |
| Bias in measurement of outcomes | Could the outcome measure have been influenced by knowledge of the intervention received? Were outcome assessors aware of the intervention received by study participants? Were the methods of outcome assessment comparable across intervention groups? Were any systematic errors in measurement of the outcome unrelated to intervention received? |
| Bias in selection of the reported result | Is the reported effect estimate likely to be selected, on the basis of the results, from multiple outcome measurements within the outcome domain? Is the reported effect estimate likely to be selected, on the basis of the results, from multiple analyses of the intervention-outcome relationship? Is the reported effect estimate likely to be selected, on the basis of the results, from different subgroups? |

Responses: Low risk, Moderate risk, Serious risk, Critical risk, No information

**Appendix 3. GRADE evidence profile template**

| **Certainty assessment** | **Number of studies** | **Study design** | **Risk of bias** | **Inconsistency** | **Indirectness** | **Imprecision** | **Other considerations** | **Effect size (95% CI)** | **Certainty** | **Importance** |
| --- | --- | --- | --- | --- | --- | --- | --- | --- | --- | --- |
| Outcome 1: [Stress measure] |  |  |  |  |  |  |  |  |  |  |
| Outcome 2: [Burnout measure] |  |  |  |  |  |  |  |  |  |  |
| Outcome 3: [Secondary measure] |  |  |  |  |  |  |  |  |  |  |

CI: Confidence interval

Certainty ratings:

- High: Very confident that the true effect lies close to that of the estimate of the effect
- Moderate: Moderately confident in the effect estimate; the true effect is likely to be close to the estimate of the effect, but there is a possibility that it is substantially different
- Low: Confidence in the effect estimate is limited; the true effect may be substantially different from the estimate of the effect
- Very low: Very little confidence in the effect estimate; the true effect is likely to be substantially different from the estimate of effect

Importance ratings:

- Critical: Outcome is critical for decision making
- Important: Outcome is important but not critical for decision making
- Not important: Outcome is not important for decision making

**Table S1. Characteristics of included studies**

| **Study ID** | **Country** | **Setting** | **Sample size** | **Mean age (years)** | **% Female** | **Nursing specialty** | **VR intervention** | **Comparator** | **Primary outcome** | **Secondary outcomes** |
| --- | --- | --- | --- | --- | --- | --- | --- | --- | --- | --- |
|  |  |  |  |  |  |  |  |  |  |  |
|  |  |  |  |  |  |  |  |  |  |  |
|  |  |  |  |  |  |  |  |  |  |  |

VR: Virtual reality

**Table S2. Summary of findings**

| **Outcome** | **Number of studies** | **Effect size (95% CI)** | **Heterogeneity (I2)** | **P-value** | **GRADE certainty** |
| --- | --- | --- | --- | --- | --- |
| Stress |  |  |  |  |  |
| Burnout |  |  |  |  |  |
|  |  |  |  |  |  |

CI: Confidence interval; GRADE: Grading of Recommendations, Assessment, Development and Evaluations
