# Supplementary material for: Virtual reality for stress management and burnout reduction in nursing: A systematic review protocol
Source: PLoS One. 2025 Apr 7;20(4):e0319247. doi: 10.1371/journal.pone.0319247 (PMC11975074; doi:10.1371/journal.pone.0319247)
Supplement: Attachment — Submitted filename: Response to Reviewers.docx [file pone.0319247.s004.docx]

**Response to Reviewers**

We thank both reviewers for their thoughtful and constructive feedback on our manuscript. We have carefully addressed each point and made appropriate revisions to strengthen the protocol. Below we provide detailed responses to each comment.

**Reviewer 1**

| **S.No.** | **Comment** | **Response** | **Changes Made in Manuscript** |
| --- | --- | --- | --- |
| 1. | The protocol is comprehensive and well-structured in assessing VR interventions for stress management and burnout in nurses. | We thank the reviewer for this positive feedback. | No changes required. |
| 2. | Would suggest to add more clarification on the exclusion of qualitative studies, and the rationale for limiting the review to English-language studies. | We appreciate this suggestion. We have now provided explicit rationale for these methodological choices in the revised manuscript. The English-language restriction reflects practical constraints and the fact that most VR intervention research is published in English, though we acknowledge this as a limitation in our discussion. | Added the following text to the Methods section (lines 195-198): "Qualitative studies are excluded as our primary aim is to quantitatively assess the effectiveness of VR interventions. While we acknowledge that qualitative insights are valuable, including them would require different methodological approaches beyond the scope of this review." |
| 3. | Additional detail on handling disagreements in assessments would be helpful. | We agree that more detail would improve reproducibility. | Added to the Methods section (lines 264-267): "For any disagreements in screening, data extraction, or quality assessment, reviewers will first attempt resolution through discussion, referring to the protocol criteria. If consensus cannot be reached, a third reviewer will review the evidence and make a final decision. All disagreements and their resolution will be documented in a decision log." |
| 4. | Would suggest to clarify how subgroup and meta-regression analyses will address heterogeneity to strengthen the methodology. | Thank you for this suggestion. We have expanded our description of how the analyses will address heterogeneity by adding methodological details. We have enhanced the Data Synthesis section to include: (1) sequential approach to heterogeneity assessment starting with visual inspection of forest plots before calculating I², (2) specification of the methods for meta-regression including use of restricted maximum likelihood estimation for random effects models, (3) minimum number of studies (n=10) required for meta-regression to ensure stable estimates, (4) handling of multiple outcome time points through sensitivity analyses comparing different approaches (e.g., using only the longest follow-up vs. separate analyses for different time points), and (5) plans for sensitivity analyses excluding influential studies identified through leave-one-out analyses. These additions provide clearer guidance on how we will systematically investigate and account for heterogeneity in our analyses. | Enhanced the Data Synthesis section  (lines 283-284) to specify: " We will begin by visually inspecting forest plots to qualitatively assess heterogeneity patterns before proceeding with statistical testing."  (lines 293-295) to specify: " For meta-regression, we will use restricted maximum likelihood estimation for random effects models, with a minimum requirement of 10 studies to ensure stable estimates."  (lines 304-306) to specify: " For studies reporting multiple outcome time points, we will conduct sensitivity analyses comparing results using only the longest follow-up versus separate analyses for different time points."  (lines 310-316) to specify: “We will conduct leave-one-out analyses to identify potentially influential studies and perform sensitivity analyses excluding these studies to assess their impact on pooled estimates.  To ensure transparency and reproducibility, we will provide detailed documentation of all analytical decisions, including any post-hoc analyses conducted in response to unexpected patterns in the data. The complete R code for all analyses will be made available as a supplementary file.” |
| 5. | More information on the cut-off values for defining high stress and burnout using the instruments would be good to improve clarity. | We agree this information would enhance reproducibility. | Added to the Methods section (lines 185-189): "For the Perceived Stress Scale, scores ≥26 will be considered high stress. For the Maslach Burnout Inventory, we will use established cut-offs: emotional exhaustion ≥27, depersonalization ≥10, and personal accomplishment ≤33. For other validated instruments, we will use published cut-off scores when available or author-defined thresholds with clear justification." |

**Reviewer 2**

| **S.No.** | **Comment** | **Response** | **Changes Made in Manuscript** |
| --- | --- | --- | --- |
| 1. | Consider providing more detailed step-by-step instructions for complex procedures. | We appreciate this suggestion and have provided procedural details throughout. | Detailed step-by-step procedures for: 1) Study screening process (lines 222-229), 2) Data extraction workflow (lines 232-253), 3) Risk of bias assessment (lines 256-268), and 4) Meta-analysis procedures (lines 278-295). |
| 2. | Including visual aids or diagrams might enhance understanding and implementation. | We agree that visual elements would improve clarity. | The manuscript has: 1) A PRISMA flow diagram template (Figure 1) showing the planned study selection process, 2), 2) A detailed data extraction form template as an appendix. |

We believe these revisions have substantially strengthened the protocol while maintaining its core methodological rigor. The added details and visual elements will enhance reproducibility and implementation. We welcome any additional feedback from the reviewers.
